# Advocacy is all of us: recommendations to enhance the Medical Library Association's advocacy initiatives

**DOI:** 10.5195/jmla.2022.1327

**Published:** 2022-01-01

**Authors:** JJ Pionke, Kathleen Phillips, Alyssa Migdalski, Erin M. Smith

**Affiliations:** 1 pionke@illinois.edu, Assistant Professor. University Library. University of Illinois—Urbana-Champaign. Champaign. IL; 2 kec5Q13@psu.edu, Nursing and Allied Health Librarian. The Pennsylvania State University. University Park. PA; 3 lyssamigdalski@gmail.com; 4 Reference and Instruction Librarian. Library of Congress. Washington. DC

## INTRODUCTION

The Medical Library Association (MLA)'s Rising Stars (RS) program is a year-long leadership program for a small cohort of participants [1]. Throughout the year-long program, there are monthly guest speakers and readings, assigned mentors, and a group project that investigates some aspect of MLA as an organization. This paper, originally envisioned as a report of our activities, explores MLA's advocacy efforts using a mixed methods approach to incorporate multiple voices and constituencies and provides recommendations to MLA for changes to those advocacy efforts in an effort to be transparent and accountable.

The 2020–2021 RS project revolved around advocacy. Our charge explicitly stated, “Particularly in the current climate of financial precarity for both hospitals and academic institutions, libraries and librarians are being asked to downsize, close, and cut resources. This has lasting and cascading impacts on education, consumer access to information, research, and clinical practice. As the largest professional group for medical libraries and librarians, MLA has advocacy written into its mission statement (#3). This includes helping members to advocate for medical libraries as institutions and for medical library positions” [2].

The 2020–2021 cohort was given four objectives. First, identify which library advocacy tools, resources, education, and initiatives are available, have been available, or are being undertaken by MLA. Second, conduct focus groups and interviews to identify the tools, resources, education, and initiatives in which MLA members are interested, either for professional growth or to actively help them advocate for their libraries. Third, based on the findings, propose an initiative for MLA to meet the continuing advocacy needs of the membership. Fourth, formally present both the findings from the research and focus groups and the proposal. In order to accomplish these objectives, the cohort decided to take a mixed methods approach that included an environmental scan of MLA and other library associations, a survey of MLA membership about advocacy, interviews with advocacy leaders from various library and information science (LIS) organizations, and self-selected focus groups with participants who took the membership survey to have discussions to better understand MLA member needs. We also created the required presentation to the membership at MLA's 2021 Annual Meeting and wrote this report with multiple recommendations from our findings in order to be transparent and accountable for our work.

## METHODS

We determined that we needed to gather several different types of data in order to accomplish the goals of the MLA RS project. Data gathering activities included:

- Examining the MLA website for policy regarding advocacy
- Examining other library organizations for discussion of advocacy
- MLA membership survey
- Interviews with experts in the library advocacy field
- Focus groups with MLA members who indicated interest in the membership survey

We used the human-centered design approach to assess survey and interview data by identifying common themes and coding them in Padlet, which allowed for long-distance collaboration during synchronous and asynchronous work. Human-centered design is a design theory that encompasses the human experience into design. It differs from user experience or accessible design in that human-centered design focuses on how human experiences interact with and change design [3]. This phase of the project continually built upon itself. The survey and interview responses added insight to the structure of, and discussion questions used, in the focus groups sessions. The information gathered during focus group discussions and the feedback received after the meetings created a clear picture of what MLA membership needs to be better advocates.

For the interviews, a list of advocacy experts from the health information professionals (HIP) and LIS fields were identified through the environmental scan. Individuals considered advocacy experts included toolkit authors and current and former leaders of committees, initiatives, or organizations centered around advocacy or governmental relations. Ten experts were invited to participate in one-on-one, semistructured interviews, half from the HIP field and half from the broader LIS field. Eight interview questions were developed by the RS team to gain a general understanding of how the interviewees approach library advocacy and how these approaches may benefit MLA. Interviewees were asked to describe how information professionals can become better advocates, offer best practices for developing and implementing advocacy initiatives, identify gaps in current advocacy efforts, and explore future directions for HIP- and/or LIS-related advocacy. Consent was obtained from each interviewee by either receiving an electronic copy of a signed consent form or receiving verbal consent before the interview took place. Two members of the team completed interviews with seven individuals. Two members of the research team analyzed the results from the expert interviews using an abbreviated thematic analysis method described in IDEO's Field Guide to Human Centered Design [4].

We determined that asking for feedback from MLA members was an important part of data collection. Therefore, we created a survey to solicit feedback from the membership about their experiences with advocacy, what they wanted help with, and how that help should be provided. The membership survey was administered using the Survey Monkey platform and ran for two weeks from November 9, 2020, to November 20, 2020, with reminders sent out every few days to encourage participation. There were 340 respondents.

Survey respondents were invited to participate in a focus group by expressing their interest at the conclusion of the survey. Thirty-two of the 340 survey respondents (9%) indicated their willingness to participate. In January 2021, emails were sent to these respondents with information about how to sign up for a focus group. Fifteen individuals (47%) participated in three focus group sessions hosted via Zoom in February 2021. All participants received a consent form with details on the purpose and procedures of the focus groups. Participation indicated consent. One focus group was reserved for academic librarians, one for hospital librarians, and one was open to any type of librarian. During each focus group, an RS team member served as facilitator, and at least one other team member took notes. The focus groups were also recorded for additional analysis. Focus group questions were developed based on responses received from the survey and expert interviews. Follow-up questions were asked when necessary to gain further insight into the participants' responses. The focus group session results were analyzed using an abbreviated thematic analysis method as with the expert interviews [5].

## RESULTS

### Environmental scan

#### Medical Library Association

Our environmental scan of MLA's website (https://www.mlanet.org/) found a number of advocacy-related resources, which included informational web pages and toolkits [6,7,8] as well as programs designed to actively engage MLA members in advocating for our profession, our values, and our patrons [9,10]. In addition, MLA has a Governmental Relations Committee [11], which articulates MLA's position on legislation as well as monitors legislation that is of interest to MLA membership, among other tasks. MLA also participates in the Joint MLA/Association of Academic Health Sciences Libraries (AAHSL) Legislative Task Force [12]. Both MLA and AAHSL work together in the task force to create a stronger and more unified front around the legislative issues that are most important to both memberships [12]. The task force also comes together to lobby various congressional bodies [12].

MLA provides a fairly robust series of webpages dedicated to providing information about the importance of HIPs through the Value of the Health Information Profession section of the MLA website [7]. This subset of webpages includes background information on HIPs and MLA, research and statistics intended to help illustrate the value of HIPs to health care providers, and strategies to communicate these messages to hospital administrators. The Values 2 ToolKit, originally developed by 2014–2015 MLA Rising Star Roy Brown, takes hospital librarian-focused advocacy a step further by “serving as a ToolKit for hospital librarians and other information professionals who need resources to plan out a library space, advocate for themselves, or improve their skills'' [6]. The Hospital Library Caucus appointed a Hospital Library Advocacy Toolkit Task Force to update the Values 2 ToolKit by fall 2021.

MLA also provides the Public Policy Center, another series of webpages aimed to enhance advocacy efforts [8]. These webpages offer information and news updates about key legislation, as well as action alerts to notify MLA members about opportunities to reach out to legislators. This year, the Public Policy Center also features details about the MLA 2021 Capitol Hill Meetings [9]. This program, coordinated through the Governmental Relations Committee, supports MLA members in engaging in successful meetings with their elected officials by providing information, tools, and resources.

Another advocacy project, the InSight Initiative, is a thought-leadership initiative designed to build goodwill and collaboration between the health sciences librarian and health information provider communities [5]. Each year, InSight hosts several summits to facilitate open dialogue about pressing issues in scholarly communications, set practical outcomes to address these issues, and allow for collaborative group work to meet the established goals. Since 2018, InSight has hosted six summits and developed a series of short videos to help information professionals better serve their users.

Although MLA offers a robust array of advocacy-related resources, discovering those resources on the MLA website is a challenge. Although advocacy is featured as one of the options on the ever-present horizontal menu located near the top of each webpage, there is no single advocacy landing page. Instead, users must select a subcategory of advocacy, such as Public Policy Center or Health Information Profession, then continue to navigate through a series of pages linked together through a secondary vertical menu. Even within the advocacy webpages, some information is either missing or outdated.

For example, the Values 2 ToolKit can only be found if one joins the Hospital Library Caucus and can therefore access the caucus community pages.

#### Other LIS organizations

We also explored advocacy offerings from MLA chapters and other LIS organizations. Organizations examined include the American Library Association (ALA) [13], Association of College and Research Libraries (ACRL) [14], Health Science Information Consortium of Toronto (HSICT) [15], Network of the National Library of Medicine (NNLM) [16], Public Library Association (PLA) [17], and Special Libraries Association (SLA) [18]. Each organization offers a unique blend of advocacy offerings, with toolkits and subgroups for advocacy, policy, and governmental relations. A breakdown of advocacy offerings by type is shown in Table 1. This table is not exhaustive, as many organizations offer unique programs, such as MLA's InSight Initiative [5] and ALA's Policy Corps [19]; however, it aims to capture the general trends in advocacy across LIS organizations.

**Table 1:** Advocacy offerings from LIS organizations

Organization	Advocacy/policy/governmental relations groups	Performance measurement	Research on value of libraries	Public awareness	Toolkits
MLA	Governmental Relations Committee [11]	n/a	Research and Statistics [20]	National Medical Librarians Month [21]	Values 2 ToolKit [6]
ALA	Public Policy and Advocacy Office [22]	n/a	Libraries Matter [23]	Libraries Transform [24]	Library Advocate's Handbook [25]
ACRL	Governmental Relations Committee [26]	Project Outcome for Academic Libraries [27]	Value of Academic and Research Libraries [28]	n/a	The Power of Personal Persuasion, Libraries Transform Toolkit [29]
HSICT	n/a	n/a	n/a	n/a	Library Value Toolkit [30]
NNLM	Varies by region	n/a	n/a	n/a	Hospital Librarian's Power Toolkit [31]
PLA	Advocacy Interest Group [17]	Several initiatives [32], including Project Outcome [33]	n/a	n/a	Campaigns That Made a Difference [34]
SLA	Public Policy Advisory Council [35]	n/a	n/a	Public Relations Advisory Council [36]	Advocacy Toolkit [18]

### Expert interviews

#### Thematic analysis results from the expert interviews

Three major themes emerged: relationship building and advocacy, showcasing the value of HIPs, and strengthening interpersonal advocacy skills of librarians.

##### Relationship building and advocacy

Relationship building was a topic addressed by many of the expert interviewees, most of whom said that relationship building was key to building effective advocacy efforts. “It's all interpersonal,” one interviewee said. Another interviewee added that successful advocacy comes from being strongly integrated into the hospital or academic system. “I'm a connecting agent for so many things,” the interviewee said. “You end up having a lot of institutional memory about projects and initiatives. [ … Librarians] really need to talk up that piece of [being integrated into the] institutional memory; you are a data storehouse. You may be the only person who knows where the bodies are buried.”

In stark contrast, one interviewee lamented the amount of focus librarians place on relationship-building skills in advocacy efforts. “Relationship building isn't enough,” this interviewee said. Instead of improving relationship skills, this interviewee suggested HIPs lobby their state and federal legislators to pass policies that mandate hospitals to employ HIPs. “I've been around a while, and I've seen a lot of things being tried and failed. It's not enough for us to do relationship building within our organization; we have to think beyond that. Now, we need to take that outside of the library and go to our legislators.” In the past several election cycles, the interviewee noted the power that policy holds in protecting library jobs. When a hospital system needs to make budgetary cuts, the interviewee said, they bring in outside people who are emotionally removed from the situation to make the cuts. “It's not enough to build relationships with the executives—when [the hospital] cuts, they cut.”

##### Showcasing the value of librarians

Many of the interviewees noted that the work of HIPs is how librarians show their organizational worth and that the work is powerful evidence for advocacy efforts. “How much money have you saved the organization?” an interviewee asked, to highlight the impact of the HIP's work on the organization's bottom-line. Interviewees also noted the importance of linking the work of HIPs to outcomes. Showcasing the work of HIPs and tying it to measuring organizational goals can elevate both the work itself and the role of HIPs in completing it successfully. “Share what you have accomplished, not just what you are going to do,” one interviewee noted. Another interviewee questioned how HIPs can move beyond the service label and into roles as invaluable partners and colleagues. “Librarians are experts,” one interviewee said; “show off your expertise; don't just serve. Help extend organizational successes or fix organizational problems.”

##### Strengthening interpersonal advocacy skills of HIPs

Most of the interviewees provided suggestions about how to strengthen the interpersonal advocacy skills of librarians and information professionals. Some characterized librarians as being stereotypically quiet, unassuming folks who are uncomfortable with selling themselves. They mentioned that practicing elevator pitches and crafting engaging stories can help propel “the ask.” Others suggested that librarians frame asks with the needs of others, other departments and other administrators, in mind. Lastly, one interviewee suggested that you “don't ask for permission; ask for support.”

### Membership survey

The top three work locations for those that responded to the survey were college/university libraries (55%), hospital libraries (28%), and specialty health libraries (5%). The respondents also indicated their position: librarian/informationist (60%), director/dean (21%), or manager (15%).

When respondents were asked to rank the advocacy challenges facing their library, the two highest ranked challenges were “don't know how to articulate the value of my library to my institution's administration or board” (31%) and “don't know how to advocate for needed resources for my library from my institution's administration or board” (31%). Other respondents selected “don't know how to advocate for budgets or budgetary assistance from my institution's administration or board” (27%) and “have never thought about library advocacy before” (20%). There was also an “other” category with 108 responses, which mostly dealt with communication issues, advocating on a political level (e.g., local, state, federal), and creating opportunities for advocacy, as well as other issues including budgets and salaries.

When respondents were asked to rank the advocacy challenges facing them as individuals, the highest ranked challenge was “don't know how to advocate for myself within the larger organization” (31%). Other respondents selected “don't know how to advocate for my role in the organization (30%), “don't know how to advocate for myself within the library” (29%), and “have never thought about advocacy for myself before” (21%). There was also an “other” category with forty-seven responses, which mostly dealt with already knowing how to advocate, communication issues, needing support, and budgets.

When respondents were asked how MLA can assist with their advocacy efforts, they selected the following canned responses, developed based on internal group discussion and overarching themes from examining other library organization websites, in order of highest importance:

1. Training on advocating for the library to organization administrators—63.22%
2. Training on how to interact with organization administrators—61.40%
3. Advocacy kit or guide—58.97%
4. Training on applying for grants—41.34%
5. Advocacy mentoring—37.08%
6. Training on how to interact with political officials—27.39%

There was also an “other” category with forty-one responses (12.46%), which mostly recommended that MLA provide examples and case studies, advocate to multiple groups such as different congressional leaders and other library associations, and provide training on advocacy for a variety of topics including budgets and diversity.

Finally, when respondents were asked about their preference for different forms of advocacy training, they most commonly selected webinars (66%), blended instructor-led courses (51%), and a track at MLA annual meetings (57%). There was also an “other” category with forty-five responses related to self-paced courses, book/journal clubs, and low-/no-cost options.

### Focus groups

#### Top issues for advocacy

When asked about the top issues for which participants typically advocate, a wide variety of topics were discussed, including funding, equal access to resources, better integration into the curriculum, proving one's expertise, providing unique user experiences, finding a voice as a nonadministrator, and raising awareness of medical libraries to members of Congress. Advocacy relating to funding and budgets was frequently discussed by many different types of librarians. One participant stated, “It's not about the money, but it's about the money.” Proving one's expertise was a major problem among academic librarians. One participant voiced their frustration by saying, “I've been a librarian for many, many years and it's getting kind of tedious to have to face the same battle over and over again.”

#### Preferred advocacy methods

Participants shared many different tactics for advocating, including elevator pitches, face-to-face appeals, doing the work of librarianship, and amplifying advocacy efforts through library champions. Some participants shared specific messages they utilize. One declared, “The library is essential infrastructure to the whole academic endeavor.” Another participant shared a similar point that the library is a “core facility,” like a magnetic resonance imaging machine. Analogy was used again to compare the library to Switzerland, as one participant noted, “We're the neutral space on campus because we support everybody on campus and beyond.” One participant appealed to humor with “Don't Google your symptoms, go to MedlinePlus.” Indirect tactics are also an option, as a participant pointed out that “doing the work for administrators and doing it well is the best advertisement for having a librarian,” a sentiment that was shared by hospital, academic, and government librarians.

#### Obstacles and frustration points

Two separate questions addressed what gets in the way of advocacy efforts and what frustration points participants face when advocating. Responses to these questions often overlapped, with participants expressing similar sentiments to both prompts. Lack of time is a problem that plagues academic and hospital librarians. Organizational politics also cause issues for both groups, with one academic librarian, who also engages in congressional advocacy, exclaiming that organizational politics are often “more political than the literal politics of Congress.” A subset of participants were particularly frustrated with how to navigate organizational politics in nonadministrative roles without “stepping on toes.” Another subset of participants struggled to effectively advocate for health sciences libraries within broader university library systems because of frustrations with “having us understand what they [non-health sciences academic librarians] do and them understanding] what we do.” Communication obstacles also impeded many advocacy efforts. These included not being able to or knowing how to access communication channels and not knowing how to effectively deliver the message. One participant shared, “It's a constant repetition. It's never going to be a one and done.” This statement could also apply to constant turnover in hospital administration, which is an issue faced by several hospital librarian participants.

#### Assistance from MLA

Participants shared a wide variety of ideas for how MLA could assist them with overcoming these challenges. There were many calls for MLA to exert advocacy efforts at the national level through a contracted lobbyist, getting a seat at the table with other organizations, such as the Association of American Medical Colleges (AAMC), American Hospital Association's Center for Healthcare Governance, and The Joint Commission, or by fighting to establish a legal mandate for hospitals to employ librarians with master's degrees. MLA has employed a lobbyist in the past, but looking forward, working with new leadership, and addressing specific membership concerns would allow MLA to renew this practice with a reinvigorated mission and vision. However, one librarian countered that they would like hospitals to decide they need a librarian instead because, “This is what you need to be a good hospital. This is what you need to be on par with everybody else.” Marketing medical librarianship at the national level was also offered as a suggestion for how MLA could help. “I would like MLA to actually advocate for all of us, use their social media accounts, build a name for themselves as an association nationally. [ … ] Why aren't we as well known as APHA [American Public Health Association]? Why aren't we as well known as AAMC?” Several participants also expressed a need for “a better body of evidence that points to the importance of librarians that we can use as a weapon in our toolkit.” It was suggested that MLA-sponsored or commissioned studies would be ideal because of the expensive, challenging, and time-consuming nature of this type of research. There were also calls for an advocacy toolkit and for “advocacy liaisons” for the MLA chapters.

Participants were also asked what form MLA's assistance should take. Several participants requested transparency for “how they [MLA] are advocating for us as a profession and as an association” There were also calls for changes to the MLA website, including “unpasswording” more content and improving the advocacy section of the website. Focus group participants wanted support from fellow MLA members, perhaps in the form of an advocacy caucus, a community of practice, mentorship, or simply more conversations like the focus groups. Shareable content was also solicited, such as things that can be retweeted or posted on social media. One of the focus groups specifically requested advocacy from library champions, “clinicians of any sort,” in “public service announcements, commercials, videos, however, whatever” created by MLA for easy reuse by health sciences librarians.

#### Most important thing

When asked which of the topics covered was of most importance to participants, there were several areas of consensus. The only item mentioned by at least one participant in each focus group session was some form of advocacy support group for MLA members. “This sort of consultation is just amazing. I don't know about everybody else, but I have never had this opportunity before. And to be in such a group and hear other opinions is just so valuable.” To further drive this point home, a librarian based outside the United States woke up at 4:00 a.m. just to attend a focus group session. The library champions video also gained some traction, with all seven participants in one focus group agreeing that the video could be extremely useful. Several participants in another group noted that developing an advocacy strategy for MLA should be a priority “before we start throwing things out there.” Finally, advocacy to other health care organizations and training resources for nonadministrators were also requested.

## DISCUSSION

The bridge between the collected data and our recommendations can be summed up in three words: vulnerability, voice, and value. MLA members are struggling in a variety of advocacy areas. One common theme among the survey, expert interviews, and focus groups is that medical librarians are experiencing vulnerability in their professions, both due to the pandemic and external factors that have existed for years. Professional vulnerability includes finding footing with administrations, advocacy timing, funding, and the impact of local and national politics on medical librarians. These factors tie directly into the theme of finding and bolstering the voices of individual medical librarians and of MLA as an organization. MLA's advocacy work has laid the foundation, but stronger action is necessary to strengthen the profession. MLA should be a more present, highly visible organization within groups beyond the library community. Finally, MLA can reinforce members' voices and mitigate issues of vulnerability through strong, ongoing, and strategic demonstration of the value of medical librarians. The message of the value should be illustrated both within and external to MLA; MLA should have many seats at many different tables and value should be central to their message.

Based upon these findings and common themes, we recommend that MLA act on and follow through with several advocacy initiatives. These include:

- create an Advocacy Caucus to build upon the advocacy work MLA has already established;
- conduct research on the value of HIPs;
- establish a national advocacy agenda to protect jobs through coordinated campaigns; and
- develop a public awareness campaign for health sciences libraries and librarians to increase public understanding of their value.

Such actions will address current and future membership concerns of vulnerability, voice, and value.

### Advocacy Caucus

One major theme that was not identified during the survey or the official focus groups but came out during informal communication during and after the focus groups was the desire to have more focused conversations about advocacy. MLA members expressed a desire for an Advocacy Community of Practice (COP). One participant said that they had never had the opportunity to participate in such a focused conversation about anything medical library-related, let alone advocacy, and expressed the desire for MLA to initiate similar discussions more regularly. COPs are “group[s of] people who share a concern or a passion for something they do, and learn how to do it better as they interact regularly” [37]. MLA caucuses, in turn, are “groups of members who coalesce around major themes of long-term concern to the membership [and] … around specialized or topical themes, [sharing] information with each other to educate, strategize, and further the aims of the Association” [10]. The overlap between an official COP and the MLA caucus is clear. Given MLA's size and global membership, we recommend the creation of an Advocacy Caucus. The creation of a new caucus would provide the platform for members to discuss advocacy issues in real time and host formal webinars, workshops, and MLA annual meeting presentations. MLA members could use the further recommendations in this paper as a blueprint for action. MLA is driven to *Build a Better Future,* a strategic goal that revolves around continuing the rigorous development of the association as a leader for HIPs [6]. The creation of an Advocacy Caucus would meet the established Education and Communities strategic goals of MLA that are not yet completed. This caucus would extend the advocacy initiatives that MLA already has in place and provide an avenue for academic, hospital, and special librarians to meet, collaborate, and act.

### Research on the value of HIPs

Assessment is considered a key component of librarianship in order to show value and relevancy, but as one survey respondent reflected, “Libraries collect a lot of 'statistics,' but are they the right data sources to advocate with the leadership we are interfacing with?” Determining which metrics matter can be challenging. Despite these challenges, both survey and focus group participants believe that more evidence proving the value of HIPs is essential to successfully advocating for libraries and librarians. Another survey respondent declared, “What would be most helpful is if MLA developed a list of suggested metrics and methods for data collection and some reporting templates that I could easily plug the info into.” Several other LIS organizations offer programs MLA could draw from to create a more robust body of evidence on the value of HIPs.

Project Outcome is a “free online toolkit designed to help libraries understand and share the impact of essential library programs and services by providing simple surveys and an easy-to-use process for measuring and analyzing outcomes. Participating libraries are also provided with the resources and training support needed to apply their results and confidently advocate for their library's future” [38]. The PLA launched Project Outcome in 2015 with the aim of moving library assessment from collecting patron counts to collecting “data to indicate the benefits libraries are providing their communities” [38]. Their standardized surveys also allow libraries to compare their data to other libraries across the nation. The tool was initially developed to measure outcomes in seven key areas related to public libraries, such as community engagement and summer reading. ACRL adapted PLA's model to measure academic library outcomes with the establishment of Project Outcome for Academic Libraries in 2019 [27].

AAHSL also conducts work in this arena with their publication *Annual Statistics of Medical School Libraries* in the United States and Canada. *Annual Statistics* contains data on a wide range of characteristics, including expenditures, collections, personnel, and services, for “academic health sciences libraries whose medical schools hold member or associate member status in the Association of American Medical Colleges” [39]. While AASHL's statistics provide a valuable snapshot of a subset of the libraries that MLA members serve, the data are not representative of the full MLA membership. Furthermore, *Annual Statistics* does not collect data that could help to illustrate the value and benefit that HIPs provide to their communities as an adaptation of Project Outcome could.

Therefore, we recommend that MLA establish a task force to collaborate with AAHSL, PLA, and ACRL to adapt Project Outcome to meet the evaluation needs of all types of HIPs. The task force would need to establish what metrics matter, develop standardized outcome measures for those areas, and conduct field tests prior to launching Project Outcome for Health Information Professionals.

### National advocacy agenda

In recent years, hospital systems, academic health centers, and universities have eliminated positions and reduced the full-time equivalent (FTE) of HIPs. Our data show that MLA members are worried about their job security. We believe MLA can play a pivotal role in protecting the jobs of HIPs. The organization can leverage its national presence and active membership to develop and coordinate a national legislative and policy campaign to mandate the existence of HIPs and elevate the visibility of our profession.

We heard from our expert interviews that some public libraries have adopted a strategy to lobby state legislatures to require the presence of a public librarian during library open hours. Medical and hospital librarians could advocate for similar legislative and policy strategies. Moreover, MLA can push accreditation bodies to require academic health centers and hospitals to establish libraries or information centers that are staffed by degreed librarians. We recommend that this national strategy be led by MLA, which would coordinate local and state-level advocacy efforts through its respective chapters and state members. This may require MLA to hire or contract with advocacy professionals to guide and/or coordinate the efforts and lobby members of Congress on the importance of HIPs. Focus group participants were clear that they want MLA to be more nationally visible and present in advocacy efforts. We believe that engaging in this type of work would help elevate MLA's visibility to its members and increase member value.

In a publication that introduced the findings of a task force established to study hospital layoffs and develop recommendations to support hospital librarians, former MLA President M.J. Tooey wrote that “no magic accreditation standard exists mandating the inclusion of hospital librarians and hospital libraries, nor is one likely to be developed and adopted” [40]. We respectfully disagree. We believe that a coordinated national advocacy strategy, paired with formal research on the value of HIPs, can improve the visibility of the important work that our members do, as well as help protect their careers.

### Raise awareness about the importance of health sciences libraries

During the expert interviews, one library advocate remarked, “Librarians are generally reticent to have themselves recognized for the work that they're doing.” Based on the passionate arguments for health sciences librarianship gathered through the survey and focus groups, MLA members do not seem to suffer from this reticence. Instead, it seems that MLA members struggle to make their messages heard due to lack of time to create marketing materials, little respect from those who most need to hear the messages, and frustration with constantly inventing new ways to say the same thing. Several respondents identified potential solutions to this problem, including easily shareable content for social media and other uses and videos and other materials featuring clinicians who are library champions. In addition, they want MLA to “take the helm” on marketing the value of health sciences libraries and librarians.

MLA has already begun work in this area. The InSight Initiative recently created a series of short videos addressing how users discover and access information resources [41]. Two of the videos are aimed at library users. One video helps users better understand how to search and the other explains how libraries can get users what they need even if the library does not have the needed resource. While these videos are a great start, we recommend that MLA expand their efforts beyond marketing specific library services to advocate more holistically for health sciences libraries and librarians. MLA could do this by collaborating with Libraries Transform, the ALA's campaign to “increase public awareness of the value, impact and services provided by libraries and library professionals” [24]. Libraries Transform includes two types of content: short snippets of information intended to educate the public about the merits of libraries with attention grabbing headlines, such as “because your data shouldn't be an open book,” and toolkits for librarians with graphics, key messages, and suggested activities to reinforce those messages. Libraries Transform currently features public-facing information about the importance of health literacy, as well as a Health Literacy Toolkit for librarians cocreated by ALA and the NNLM [24]. MLA could partner with ALA and the NNLM to grow the Libraries Transform's Health Literacy Toolkit to include materials on how libraries and HIPs impact patient care, medical education, and research. By partnering with ALA and NNLM, MLA can capitalize on the established success of the campaign to reach a wider audience and raise the profile of health sciences libraries and librarians.

## CONCLUSIONS

As one of the focus group participants stated, “Advocacy is all of us together, not just us as individuals.” MLA has made great strides toward the creation of new and the improvement of existing advocacy initiatives. There is no time like the present to continue this vital work for the good of the health sciences libraries profession. This work of the RS Advocacy Cohort highlights the immediate needs of the MLA membership and provides a clear path for MLA to continue forward momentum in this area.
