# Erratum to: Pionke JJ, Phillips K, Migdalski A, Smith EM. Advocacy is all of us: recommendations to enhance the Medical Library Association's advocacy initiatives. J Med Libr Assoc. 2022;110(1):5-14. DOI: https://doi.org/10.5195/jmla.2022.1327

**DOI:** 10.5195/jmla.2022.1489

**Published:** 2022-01-01

**Authors:** JJ Pionke

The name of one of the authors was misstated during the manuscript submission and publication process. The author's name Kathryn Phillips was corrected to Kathleen Phillips. The original manuscript has been updated to reflect this change.

